# The Engineering Side of Hospital Work

**Published:** 1905-11-04

**Authors:** 


### THE ENGINEERING SIDE OF HOSPITAL WORK.

### XV.—ELECTRICAL APPARATUS.

#### Two- and Three-Phase Currents.

Two- and three-phase currents are simply two and three alternate currents, having all the properties described in connection with simple alternate currents, the two and the three currents being generated by one or a group of dynamos, and the two or the three being connected in the same system, but independently of each other. With the two-phase system there are two separate sets of coils in the generator in which the separate currents are generated, and there are two distinct pairs of cables in which the currents are delivered to the motors, or whatever may use the currents. This is the usual arrangement, but sometimes three cables are employed, one for each phase, and the third acting as a return circuit for both. With three-phase currents there are three sets of coils in the generator and three cables only for delivering the currents ; but, again, four cables may be employed with three-phase working, the fourth cable being connected to the neutral point between the three sets of coils. In all cases, however, the two or the three currents must be used together. In the two-phase arrangement a pressure exists between the two cables of each pair, or between each cable and the third, where three are used, similar to that which exists between the two cables, or conductors of a single-phase system ; and a similar pressure exists between each pair of the three cables in the three-phase system, a smaller pressure existing between each of the three cables and the neutral cable. But when the two- or the three-phase system is in use, the currents passing in the different phases must be approximately the same, or there will be losses and troubles in the generator, owing to induction, similar to that described in connection with electro-magnetism. The two- and three-phase systems were first introduced because the single-phase system does not lend itself to the distribution of power. With the continuous - current apparatus, any generator is also a motor, if it is required to be so, and *vice versa.* The single-phase generator is also a motor if it is arranged to run at exactly the same speed as the generator that is furnishing the current to drive it, and if it is provided with magnetism from a source of continuous current. Two- and three - phase motors are not constructed similarly to the generators, but they do not require any special arrangement for providing the magnetic field, nor do they require to run at the same speed as the generator. Lamps, however, and other apparatus are supplied with current, when convenient, from two- and three-phase services, the apparatus supplied being divided between the phases so as to keep the currents passing in each balanced. In the two- and three- phase apparatus there is no gain of output, or of anything else, except a slight gain in the size of the cables, but there is sometimes additional convenience ; with two- and three-phase plant, the output is divided up between the two or three circuits.

#### Pressures of Alternate and Continuous-current Services.

For lighting or power, the working pressures delivered by alternate currents, whether of the single-, two-, or three-phase variety, are the same as those delivered by continuous currents. In fact, the construction and output of alternate current services are designed and measured by the heating effect produced in an incandescent lamp. A lamp arranged to work with 200 volts, for instance, and to give a certain candle power, will give it, if that pressure is applied at its terminals, no matter whether the current is continuous or alternating. In the case of alternate currents, what are termed virtual volts and virtual amperes are taken, these being the volts and amperes that will produce the same effect as the same number of volts and amperes in continuous currents ; they are referred to above as working pressures. There is a correction to this for power, that will be referred to later. And these virtual pressures are the pressures existing for practical purposes, between the two cables of a single-phase system, and between the pairs of cables in the two- and three-phase systems. But the actual pressures reached by all alternate currents used for lighting and power, are 1·4 times as great as the virtual pressures, and as the currents are reversed from 50 times a second upwards when an alternate service is applied to the human body, the difference of pressure present, at these intervals, is 2·8 times the virtual, or working pressure. This means that with a 200-volt service, the actual difference of pressure to which any body is exposed, say when clasping two conductors between which this pressure exists, in the hands, is 560 volts. With high-frequency currents, the rising and falling of the pressures, and currents is not the same as with the alternate current supply services, but the same thing holds ; the difference of pressure to which the body is exposed is very much greater than the nominal pressure of the service.

#### Apparatus for the Generation of Electricity.

With a few exceptions the apparatus employed in generating electricity for practical electrical engineering work are the same as those for medical and surgical purposes, the main difference being the fact that much smaller quantities are used for the latter purposes. Machines for generating electricity may be divided into two classes—the primary generators, those which generate electricity when driven by power or when supplied with fuel in themselves; and secondary generators, which are really transformers; they take electricity from one source and store it, or convert it, to the form required. It may be taken also as an axiom that the modern electrical engineer, if allowed to have what apparatus he wants, can convert any form of electricity, except the static form, into any other form, and that he can produce any form of electricity, including the static form, from any other.

The primary generators at present in use are:—The static induction machine, the galvanic battery, and the dynamo machine.

The static induction machine is a development of the very early experiment in which glass rubbed with silk became electrified. The early static machines consisted of glass or ebonite plates or cylinders, arranged to be rubbed by silk or other substances, and to give up the charge they acquired to the prime conductor of the machine. In the static induction machine the principle of electrostatic induction is made use of to obtain better, and larger results. The principle of electrostatic induction is well illustrated by the gold leaf electroscope, in which two gold leaves are suspended from a rod fixed in the cork of a glass bottle, the upper end of the rod projecting above the cork, and the gold leaves being inside. As long as no charge is present, the gold leaves lie together, but when a charged body is brought near the rod, the gold leaves become charged by induction in the same manner as the inducing body, the theory being that the electricity of the same name is driven into the gold leaves, charging them and causing them to diverge. In the static induction machine two or more glass or ebonite plates are arranged to revolve, power being employed, usually an electric motor in the larger sizes. A small charge given to one of the plates, is multiplied by induction as the plates revolve, the mechanical energy of the driving power being thereby converted into electrical energy, at very high pressure, but having very small quantity, and it can be taken from the machine by means of the prime conductors, two brass rods with balls attached, arranged for the purpose The static induction-machine is a generator of very high resistance, and hence the quantity of energy it transforms is very small, notwithstanding the fact of its very high pressure. This accounts also for the phenomena that sparks of comparatively large size may be taken from the machine by the human body, without danger. When the apparatus is delivering sparks it is furnishing current, just as a dynamo does when driven, though the form of the current is so different that the fact is masked. The latest form of machine is that made by M. Gaiffe of Paris, and sold by the Medical Supply Association of Gray's Inn Road, in which a number of ebonite plates are used.

*(To be continued.)*

